# Taking Situatedness Seriously. Embedding Affective Intentionality in Forms of Living

**DOI:** 10.3389/fpsyg.2021.599939

**Published:** 2021-04-23

**Authors:** Imke von Maur

**Affiliations:** Department of Philosophy of Cognition, Institute of Cognitive Science, Osnabrück University, Osnabrück, Germany

**Keywords:** situatedness, affective intentionality, practice, form of living, habit, affective biography, socially extended mind

## Abstract

Situated approaches to affectivity overcome an outdated individualistic perspective on emotions by emphasizing the role embodiment and environment play in affective dynamics. Yet, accounts which provide the conceptual toolbox for analyses in the philosophy of emotions do not go far enough. Their focus falls (a) on the present situation, abstracting from the broader historico-cultural context, and (b) on adopting a largely functionalist approach by conceiving of emotions and the environment as resources to be regulated or scaffolds to be used. In this paper, I argue that we need to *take situatedness seriously*: We need (a) to acknowledge that emotions are not situated in undetermined “contexts” but in concrete socio-culturally specific practices referring to forms of living; and (b) to agree that not only are context and emotions *used* for the sake of something else but also that the meaning-disclosive dimension of affective intentionality is structured by situatedness as well. To do so, I offer a multidimensional approach to situatedness that integrates the biographical and cultural dimensions of contextualization within the analysis of situated affective dynamics. This approach suggests that humans affectively disclose meaning (together) which is at once product and producer of specific forms of living – and these are always already subjects of (politically relevant) critique.

## Introduction

A political caricature might amuse one person, leave another unmoved, give rise to outrage in another, and prompt thoughts of murdering the caricaturist in a fourth. Some people feel pure anger while filling out a form offering a third box between ‘‘male’’ and ‘‘female,’’ while others feel relief when ticking that box. The release of the newest Thermomix elicits great excitement in many, whereas others can only shake their heads about this way of ‘‘cooking,’’ while a few might exist who cannot but be indifferent about this, because they do not even know what a Thermomix is. These cases are not abstract and sterile examples from and for textbooks. They are ways in which humans affectively disclose meaning and thereby do not only make up their own worlds, but the worlds of other humans as well. If trans persons are confronted with hate, disrespect and even the denial of their identities and rights; if, on a societal level, the practice of cooking gets lost because whole cultures following ‘‘food trends’’ lose the capacity of that craft; if a teacher gets beheaded because of discussing Muhammad caricatures in class, 5 years after journalists were murdered for publishing one in Charlie Hebdo -- the emotions involved in such kinds of world-making need to be understood and evaluated. But how can these emotions or the absence of such be explained and how can we assess which affective reaction is appropriate? The thesis of the present paper is that there are emotions which can neither be understood nor be normatively assessed without reference to what I call ‘‘forms of living’’^1^. Setting out this thesis, I *take seriously situated approaches* to emotions in this paper and develop a *multidimensional approach to situatedness*.

The “situatedness paradigm” of affectivity can be seen as a similarly influential refocusing like the “cognitive turn” within both the psychology and the philosophy of emotions in the 1960s. The framework of situatedness, which has already been well established for cognitive processes (Robbins and Aydede, 2009 and Newen et al., 2018 for an overview), got transferred to the affective realm (Wilutzky et al., 2013; Stephan et al., 2014; Krueger and Szanto, 2016 or Stephan and Walter, 2020 for an overview): Emotions are no longer regarded as purely private affairs of an isolated subject, but as phenomena which are inevitably contextual. Instead of focusing on individual agents and unidirectional episodes of emotions (a particular emotion type being directed at a concrete object), situated accounts investigate affective phenomena which unfold *between* individuals and their social and material environments in dynamic processes.

The impetus of theories of situated affectivity – namely overcoming an individualistic or even intrapsychic paradigm of emotions – is of great import. Yet, while the emphasis on the significance of embodiment and environment for understanding affectivity is right and necessary, the pioneering situated approaches in the philosophy of emotions which provide the frameworks and conceptual toolbox for analyses do not go deep enough. They focus on the impact of body and environment on single affective episodes in a concrete moment while abstracting from the broader socio-culturally and historically specific biographical context (e.g., a jazz musician who regulates their emotions by means of their instrument or a marital quarrel in a given social setting; see Griffiths and Scarantino, 2009; Krueger, 2014; Stephan et al., 2014; Colombetti and Krueger, 2015). Additionally, these accounts mainly focus on the functional aspect of situatedness, viewing emotions as “to be regulated” and the environment as “to be used” (see Slaby, 2016 or Stephan and Walter, 2020, who call this the “user-resource-model”). What is missing is a conceptualization of the situatedness of *affective intentionality as disclosing meaning*: that humans represent their surroundings as being meaningful in a specific sense by means of their emotions. Or as Wittgenstein famously has it: “The world of the happy man is a different one from that of the unhappy man.” Taking these two restrictions together, what is missing in the work on situated affectivity is to provide a conceptual framework for this affective way of disclosing meaning in its situatedness within socio-culturally specific practices. To be able to analyze this is of utmost importance for understanding and normatively assessing urgent and prominently discussed affective phenomena with political relevance like the ones mentioned above.

My multidimensional approach to situatedness integrates the concrete situation of affective dynamics within a broader context. Based on the assertion that it is not “context” (as an abstract variable) in which affective processes unfold but *concrete* socio-culturally and historically specific *practices* and *forms of living*, I argue that the specificity of such a practice and form of living systematically structures the characteristics and the content of emotions. To acknowledge this, we have to look beyond the concrete moment in terms of both time and space – we need to consider the affective biography of the feeling person as a product and producer of the specific ways in which body and environment affect the way in which emotions disclose realities. Without acknowledging this, we cannot adequately explain why certain affective processes unfold in the first place, how they are experienced, interpreted by the self and understood or even sanctioned by others and how to assess their appropriateness. The framework I develop aims at enabling an assessment of life-form specific structuring effects of *situated affective intentionality* – and, if necessary, at a politically relevant critique of situated affective intentionality. The aim of the paper is to open a new perspective for a politically engaged philosophy of affectivity. As such it provides an overview of, and wants to motivate, a new paradigm of situated affectivity. Achieving this aim requires that relevant aspects and analyses of single cases cannot be discussed in all details and depths – this paper rather is meant to offer a framework for such.

In the section ‘‘Affective Intentionality in Life-Form Specific Practices: ‘Little Worlds,’’’ I introduce the concept of ‘‘little worlds’’ to denote the context in which affective intentionality is situated as structured by concrete practices which refer to forms of living. To denote the content humans disclose via affective intentionality, I introduce the term ‘‘meaningful Gestalt.’’ This content is only intelligible against the background of the practices and forms of living which again make intelligible the ‘‘little worlds.’’ In the ‘‘Situatedness I: Synchronic-Local Perspective’’ section, I adopt what I call a ‘‘local-synchronic’’ perspective on affective intentionality. This means looking at the present moment, at concrete affective dynamics between individuals and/or the material environment and the impact of such contextual factors on the characteristics and content of affecting and being affected. Importantly, I conceive of the context and the emotions not in functionalist terms but (a) in terms of meaning disclosure and (b) in their practice-specificity. In the ‘‘Situatedness II: Diachronic-Global Perspective’’ section, I adopt a ‘‘global-diachronic perspective,’’ i.e., I focus on the intertemporal dimension of life-form specific embeddedness -- the ‘‘affective biography’’ of an individual. Additionally -- this is the ‘‘global’’ feature of that perspective -- I consider socio-cultural factors which lie beyond concrete local, present moment affective dynamics, namely encompassing historical and societal structures such as emotional fashions, ideologies or regimes tacitly shaping the present moment dynamics. While in the first two sections the individual is situated within a context, in the section ‘‘Situatedness III: Forms of Living Within the Subject: Normative Assessment of ‘Little Worlds’’’ I invert this perspective and situate the life-form specific context within the feeling individual. To adopt this perspective is a consequence of my conviction that it is not sufficient to put ‘‘naked’’ subjects into a context and *afterward* analyze the effects of such a contextualization on the characteristics and content of the involved feelings. Rather, what the multidimensional situatedness framework of this paper indicates is that life-form specific situatedness structures the space of possibilities for affecting and being affected as well as the content and characteristics of actual affective engagements in a much more fundamental way^2^. Importantly, the historico-social, biographical (diachronic) and inverted dimensions I develop are not optional “add-ons” which can *also* be considered when thinking about situatedness. Rather, they necessarily structure the synchronic local perspective at issue, in the approaches providing the conceptual toolbox for situated affectivity – this is what is meant by “taking situatedness seriously.” This shift in perspective has also serious consequences for a normative assessment of emotions. What we ultimately evaluate when we deem concrete ways of affective disclosure to be (in)appropriate are the forms of living they enable, sustain or prevent.

## Affective Intentionality in Life-Form Specific Practices: “Little Worlds”

Emotions, according to the core assumption of situated approaches to affectivity, are not private affairs but embedded in or even extended by the socio-material environment. This insight is of great import. Yet, the frameworks and concepts for situated approaches to affective phenomena do not go deep enough in addressing specific ways of affective reality construction with political relevance. This restriction can be revealed by considering the two main ways of addressing the relationship between the feeling person and environment offered so far: (1) to conceptualize emotions as strategies for manipulating the environment (cf. Griffiths and Scarantino, 2009; Wilutzky, 2015) and (2) to focus on emotion regulation through an active manipulation of the environment (*scaffolding* and *niche construction*; Krueger, 2014; Colombetti and Krueger, 2015). A paradigmatic example for the first way is a marital quarrel in which emotional expressions are used to test how the other one reacts – to get information about the context (Griffiths and Scarantino, 2009; Wilutzky, 2015). The second way concerns the active manipulation of one’s emotions by *making use of the material environment*, for instance by listening to specific music or going to a certain place such as a church versus a sports event (Colombetti and Krueger, 2015). In both ways, emotions and environment are (i) considered regarding their *functional* aspect – emotions as strategies or a resource to be regulated, and the environment as a functional niche or scaffold. And (ii) their situatedness primarily concerns the present perspective of concrete affective encounters in a given environment.

But emotions and environments are not only *used* for the sake of something else (epistemic, pragmatic, or regulative purposes) but structure the very space of possibilities in which *meaning* is disclosed by self and others. This (shared) disclosure of meaning takes place in a concrete situation, yet the *specificity* of this situation and *how* the contextual factors shape the affectively disclosed meaning is only understandable against the background of specific practices and forms of living. In order to understand how humans – *as* beings engaging in socially shared practices and living specific ways of life – disclose meaning (together) affectively we need concepts which denote this practice-relatedness for both, the meaning disclosed and the situational context being producer and product of such affectively disclosed realities. I call the former “meaningful Gestalts” and the latter “little worlds” and introduce them now before I can establish the multidimensional situatedness framework in the sections afterward.

In the same way as I build upon the framework of situatedness, I take for granted the insights of the work being done on *affective intentionality*, namely that via emotions humans disclose something about themselves and the world (see Goldie, 2000; Roberts, 2003; Slaby, 2008 among others). But it is crucial to clarify how I understand emotions and their content in the following. The content disclosed via emotions, namely their presenting the self and the world as meaningful in specific ways (as opposed to being merely internal physiological arousals) is what I call *a meaningful Gestalt.* With this concept I reject the idea that emotional content is reducible to well definable evaluative properties like “the dangerous” or “the beautiful” – what is called the formal object of an emotion. Based on the insight that this alone does not specify the concrete content of emotions well enough, Bennett Helm (2001) introduces the helpful concept of *focus* to the debate of affective intentionality to denote the background concern which makes intelligible the formal object of an emotion in the first place. This brings out the reasons for why I am afraid of an angry looking crowd passing my bicycle on the street – namely the meaning it has to me and my desire for it to remain intact. The occurrence of a specific type of an emotion in a specific situation (here: fear) is only understandable with reference to a more encompassing pattern: what is disclosed via emotions is embedded in a net of concerns and meanings of the subjects going beyond the present moment. I would also feel relief accordingly if the crowd just passes without even noticing my bike. Robert Roberts (2003) adds to this picture the concept of emotions as *concern-based construals*. Similar to how we visually perceive Gestalts in pictures for instance (like the Wittgensteinian duck-rabbit), we at the same time receive certain input and construe its meaning. This is why I understand “disclosure” not as a merely receptive term but as performative as well. It is not only one single aspect but a whole meaningful Gestalt that is brought into existence when we feel in certain ways, not only for us, the feeling person, but for our environment as well. When a teacher is ashamed because they made a mistake in a lecture they do not only privately experience the situation as shame-worthy. They also construe a “reality” being shared with the students. This reality or: “little world” – as I call it and introduce in a moment – provides the space of possibilities for other affective reactions following the teacher’s shame from their side as well as from the students’. The reality – the context – is a different one than if the teacher would have reacted with laughter. And this Gestalt they, as individuals, are aware of by means of their lived bodily experience (for a detailed version of this account of affective intentionality see von Maur, 2018, chapter 2).

The notion of a “little world” refers to Lugones’s (1987) introduction of ‘‘worlds’’ to denote multiple ways of being and the navigation with and between them from a phenomenological perspective^3^. For instance a person might inhabit the “world” of academia, the particular idiosyncratic world of their family, of being a woman in a male-dominated workplace or that of “being a Latina.” These “worlds” are experienced differently and demand different kinds of (affective) comportments. In different “worlds” subjects are more or less “at ease,” as she claims; in some worlds we are able to “sink in” (Ahmed, 2006), whereas others are burdensome or even not opened up. Importantly, humans can inhabit different “worlds” while being in the same space:

*“Both you and I might be in the same room of the same building in the same city, but if you are a white United States-born citizen and I am a Latin American born in Nicaragua, we will probably have different takes on what we experience in this room, and we will have different takes on our experiences depending on the dominant norms and practices of the particular situation and how we relate to these practices given the contexts which dominate our particular interpretations” (Ortega, 2001, p. 11).*

I adopt the term ‘‘little world’’ to highlight this specific normativity structuring the disclosed Gestalt (with the decidedly political implications). A concrete situation in which individuals disclose meaningful Gestalts (together) is describable as such a ‘‘*little* world.’’ These can but do not have to coincide with more prevalent, enduring and dominant descriptions of society, such as gender or class, but can also be more idiosyncratic as I will later discuss, for instance the ‘‘little world’’ people disclose because of posting anything about their life in ‘‘social’’ networks. The teacher example above shows that it might be only once that a particular ‘‘little world’’ is disclosed, whereas others are enduring practices and more stable forms of living -- such as being a climate activist or a fan of a particular basketball team. A ‘‘little world’’ can be occupied by just one person, but mostly the affectively disclosed meaning and normative structure refers to something socially shared. I might disclose my low-carb-superfood oatmeal alone at home as fulfilling my need for a healthy life, but this is in its specificity only intelligible against the form of living perpetuated through media, advertisement -- i.e., a meaningful Gestalt materialized in social practices. Thus, I consider the situation in which affective intentionality takes place as a (shared) ‘‘little world,’’ that is: as a practice-specific reality (at a concrete time and place) referring to a *form of living*^4^. Accordingly, the environment an emotion takes place in not only provides the frame for sending or getting social signals, to gain information or to dampen or amplify emotions, but it essentially involves individuals in specifically meaningful realities of life. In any concrete affective dynamic, something involves and touches the subjects. These realities are not enacted by individuals alone but in shared processes with others and material factors which are always already meaningful – meaningful, that is, against the background of forms of living.

Forms of living concern the cultural and social reproduction of human life. As such they do not only express themselves in different beliefs, value orientations and attitudes, but also materialize themselves in fashion, architecture, the justice system and ways to organize families (Jaeggi, 2014, p. 21). Importantly, forms of living are not personal, private affairs: they are not individual options but “transpersonally shaped forms of expression with public relevance” (ibid., p. 22). For instance, to adhere to or refuse a gender specific behavioral order is a disposition unavailable to individuals alone insofar as it relies on socially constituted patterns of comportments and meanings. The behavior of an individual inevitably affects not only those adhering to or refusing these patterns, but it also shapes the space of possibilities of others (ibid.). A boy, according to Jaeggi, is not able to cultivate his preference for pink clothing innocently for very long without being confronted with the circumstance that – in some societies – his taste is coded as “girlish” (ibid., p. 22 fn. 7).

In order to understand what it means to address the situational context in which emotions take place *as a life-form specific* context, a praxeological perspective is of help: because any form of living finds expression in specific practices and in turn, any practice refers to a specific form of living. Practices can be understood as performances of skilled bodies which are neither reducible to mechanical movements, nor conducted in the mode of reflexively or consciously intended actions. Someone who masters a specific practice *embodies* the knowledge, the skill; it is inscribed into the lived body in a way that the life form specific comportment becomes “second nature” (Scheer, 2012, p. 202). Practices are, at one level, composed of such individual performances. Yet these take place in, and are only intelligible against, the more or less stable background of other performances. Emotions are thus situated in contexts in which humans skillfully perform practices which are in their specificity intelligible against the background of concrete forms of living. Taking situatedness seriously involves investigating the influence of this kind of contextualization on the way humans are situated affectively in what I call ‘‘little worlds’’ -- namely (shared) spaces of *complex meaningful Gestalts*^5^. In the following section, I zoom in on concrete affective dynamics to explore the life-form specific structuring effects of situatedness on phenomenal character and the disclosed content of affective intentionality in (i) interpersonal and (ii) socio-material practices.

## Situatedness I: Synchronic-Local Perspective

With an emphasis on the reciprocity, flexibility and openness of affective dynamics, situated approaches focus on the exchange of signals for the means of relationship configuration (Griffiths and Scarantino, 2009) or for epistemic purposes (Wilutzky, 2015). But the back and forth of affective interactions can also be addressed regarding the shared construction of “reality.” The social psychologist Wetherell (2012), for instance, takes into view such normative sequences of situated affective dynamics by means of conversation analysis:

*“The positions taken up are responsive to what has gone before, and are often loosely paired with each other. The affective pattern is in fact distributed across the relational field and each partner’s part becomes meaningful only in relation to the whole affective dance […] We create contexts for others as we act. Then, in reply, the other we have addressed orients to what is taking shape and remakes the context again” (Wetherell, 2012, p. 87).*

In affective dynamics, patterns develop for possible emotional reactions built upon those the dialogue partner offers, so that the “little world” and the Gestalt change likewise in a metamorphic process. This transformation dynamic is not only an interpretative framework of outside observers but is *experienced* by the involved subjects *through* their lived body. This can be described as a “sensual metamorphosis” – to use a term by sociologist Jack Katz. In his book *How Emotions Work* (Katz, 1999) Katz documents several studies he conducted about car drivers in a chapter called “Pissed off in L.A.”. The fact that the reports analyzed are from Los Angeles is relevant. Driving a car in L.A. significantly differs from driving a car (as the general practice) in other contexts – for instance on a country road or on a highway in the Rocky Mountains. Also, anyone who has ever driven a car in Italy or France knows that driving and going postal – e.g., sounding one’s horn – differs in frequency and intensity (i.e., in the affective involvement) a lot depending on the cultural setting. At first glance the scenes of outrageous car drivers seem to be characterized by the fact that they descend upon the person dramatically and unfold and progress in an uncontainable manner. This would support the common view according to which emotions are primarily (or even merely) an expression of internal physiological arousal of single individuals. But if driving the car is addressed as a bodily experienced socio-cultural practice it becomes visible that these affective processes do not develop like a chaotic hurricane but rather exhibit a specific *normative order*. Take this example from Katz:

*“Lori, who is originally from Georgia but has lived in L.A. for many years, prefers public transportation but must drive here routinely. When ‘a big new brown truck … decided to cut her off, Lori turns to the truck, ‘What do you think you are doing? You know better than that!’ She talks to herself and uses hand motions. She looks toward the driver in a sideways glance and then talks facing straight ahead … She does not want to lose her life over a driving dispute.’ But after she goes through scolding motions ‘she [can] drop it”’ (Katz, 1999, p. 19; also quoted in Wetherell, 2012, p. 77).*

Because of the *established practice*, Gestalts are offered that are “worth freaking out over” – like tailgating, flashing headlamps, etc., which lead to typical emotional reactions expressed by screams of outrage, threatening gestures and mumbled (or loudly uttered) swear words.

This structure of affective dynamics cannot be explained by the established affective style between subjects who know each other well (as the so far established situationist approaches would do), but rather stems from the *shared practice* they are involved in, and the rules and norms which are known and accepted or refused (tacitly). Think of an escalating affective tumult emerging when those wanting to enter a train systematically block the doors and nobody can leave the train, or if a passenger realizes that someone else -- supposedly wrongfully -- is sitting in the seat they made a reservation for. Here affective dynamics emerge which -- independently from the concretely involved individuals and their concerns -- reveal an astonishingly intertemporal persistence in their patterns. The normative back and forth appears to be downright *scripted*^6^. There are roles for specific affective performances in which people slip in and out like professional actors.

Not only are other people part of affective processes, but also spaces, objects, infrastructures, etc., -- the material ‘‘non-living’’ environment -- build their context^7^. Freaking out while driving the car for instance co-depends on the way in which traffic is regulated:

*“Those in cars whizzing toward us on the opposite side of the motorway or on the other side of the dual carriageway are rarely assholes. ‘Assholeness’ entirely depends on patterns of contiguity and common movement and, thus, occurs most often in relation to cars and drivers immediately in front of us and behind us heading in the same direction” (Wetherell, 2012, p. 88).*

The car itself can even be interpreted as a physical extension of the self which enables specific ways of interaction with other vehicles (or the drivers). In this line, Katz even conceives of the car as being integrated in the body schema^8^ of the driver. This could explain why primarily the drivers and not the co-drivers freak out and why driving an SUV feels different from driving a Smart. To consider cars and the contextual factors of being close or far away as material structuring factors on affective intentionality concerns how these factors impact the disclosure of another object (of the car driver, the whole situation, etc.). But also, the intentional objects are addressable from a practice-specific perspective. Here the concept of “affordances” is of help. James Gibson (1986) introduced the term for relational properties of objects which provide or prevent specific action-oriented offers -- affordances -- to the perceiver^9^. For instance, a chair is perceived as affording to be sat on or a piece of cake affords to be eaten. Making use of it for the realm of emotions, the concept of affordances concerns the phenomenological observation that some aspects in a situation have a specific “affective allure” (Rietveld, 2008, p. 977) or “affective power” (Romdenh-Romluc, 2013, p. 11) – they are felt as being salient in contrast to others and thus afford specific actions (see also Hufendiek, 2016 for a detailed approach to emotions as representing affordances). For the purpose of the present paper there is a relevant extension of Gibson’s account, put forward by Allan Costall (2012) who suggests that we differentiate between ordinary and what he calls “canonical” affordances. The latter are distinctly concerned with the *socio-cultural background of practices* which make the affordance of an object intelligible:

*“[S]uch affordances are situated not just in the ‘current’ behavior setting, but also in a more encompassing, shared and historically developed constellation – such affordances exist as they persist in shared and social practices […] They exist as many individuals act on them in more or less appropriate ways, in the totality of practices that, together with other affordances, sustain them” (van Dijk and Rietveld, 2017, p. 3).*

In line with the key assumption of my multidimensional approach, the claim is that the relevant aspects of the environment of an individual in a concrete situation are only comprehensible insofar as they are considered as part of a more encompassing constellation of practices beyond the present moment (van Dijk and Rietveld, 2017). Material aspects are thus embedded in and comprehensible against the background of a conglomerate of practices too. The ordinary understanding of materiality as “pre-formed substances” (Orlikowski, 2007) has to be reconsidered accordingly and materiality and socio-cultural practice have to be seen as constitutively intertwined:

*“[T]he social and the material are considered to be inextricably related – there is no social that is not also material, and no material that is not also social” (Orlikowski, 2007, p. 1437; also quoted in van Dijk and Rietveld, 2017, p. 4).*

The relationship between a practice and an affordance is according to van Dijk and Rietveld an example for such a relation of “constitutive entanglement”:

*“A specific practice and the affordance taking shape within it are interdependent and none of the two is prior [to] the other. Any affordance implies a practice which it realizes and any practice implies a landscape of available affordances” (2017, p. 4).*

To transfer this insight to the situatedness of affective intentionality as established so far, with a focus on the disclosure of practice-specific materiality, consider the following vignette:

*Alex enjoys the first spring sun while shopping in Berlin. They are in the capital for an internship, but right now it’s the weekend: leisure time. Alex already came across a variety of hip shops, bought trendy clothes and tested a fancy kale smoothie. While imagining, with a huge smile on their face, how to combine the new clothes and what to wear for the party tonight with their colleagues, Alex passes an impressive arrangement of gray blocks of stone. They feel the need to take a picture and share it on Instagram. A yoga pose, that would look great – Alex thinks. And in the next moment they ask a person to take a picture of them on the stone, one leg behind and the arm to the front. “Awesome!” Alex thinks happily, puts a hashtag below the picture and clicks “share.” Filled with feelings of urbanity, creativity, inspiration, and freedom and a thrill of anticipation of the many likes and comments the picture will receive, Alex continues their shopping trip through Berlin.*

How can Alex’s emotions be explained without reference to the form of living their affective disclosure represents? Which relation holds between the properties of the stone blocks and Alex’s reaction of happiness and enthusiasm? From an affordance perspective one could say that they perceive the stones as being “Instagram-able.” Adding Helm’s concept of *focus* we can specify that their happiness arises from the background concern which determines the meaningfulness of the object. But how can the background concern and the meaningfulness of the stones be described without reference to the socio-cultural practice of the very specific way of interacting on “social” media? Although it is true that these follow very specific normative rules which are permanently subject to subtle processes of change which are hard to understand for “outsiders” – there *is* something “at issue and at stake” (Rouse, 2002) that might escape being graspable by language, but that systematically structures the complex Gestalt that Alex discloses and the focus making the disclosed reality intelligible in the first place. This practice -- referring to what I call the form of living of ‘‘posting’’ -- structures the properties of a specific intentional object for different people as ‘‘post-able’’ (or Instagram-able, YouTube-able, Facebook-able, etc.), whereby the *concrete* Gestalts which are disclosed are possibly highly idiosyncratic^10^. A fashion blogger also presents the stone blocks as “post-able” because they inhabit the practice of “posting,” but a different Gestalt is disclosed – they see themself in a specific style, associated with possible advertisement partners, etc. A couple, in turn, wants to share their everyday life with “friends” on Facebook and takes a “partner-selfie” that should demonstrate (or even realize) happy moments and the narrative of the perfect relationship.

Importantly, this suggested *practice-specific affordance account* makes visible why certain objects, as opposed to others, even appear *as* objects for a certain affective disclosure – why *they* “pop-out” of a landscape of many possible affordances in that specific way (see von Maur, 2018, chapter 2.4 and chapter 4 for a detailed account on the pre-reflexive level of habitual affectivity)^11^.

The “skillfulness” of affective intentionality, which is conceptualized in action- and goal-oriented functionalist terms in other situated approaches (cf. Wilutzky, 2015; Hufendiek, 2016), thus shifts on my perspective: The skillfulness dimension refers to the ability to affectively disclose what is “at issue and at stake” (Rouse, 2002) in a given practice. To reformulate Helm’s concept of the focus from the perspective of the situatedness in life-form-specific contexts thus means to understand the concerns of the individuals against the background of what is “at issue and at stake” in a concrete situation relative to a specific practice and the norms constituting it. With this phrasing Joseph Rouse describes the normative element of practices, which is not reducible to either explicit rules or regularities, nor graspable or expressible through language.

*“[W]hat a practice is, including what counts as an instance of the practice, is bound up with its significance, in terms of what is at issue and at stake in the practice, to whom or what it matters, and thus with how the practice is appropriately or perspicuously described” (Rouse, 2002, p. 175).*

*“Our normative reach always exceeds our grasp, and hence what is at stake in practices outruns any present articulation of those stakes. […] We are accountable to what is at stake in our belonging (causally and normatively) to the material-discursive world: our fate is bound up with what is at issue and at stake in our practices, although those stakes are not yet definitively settled – indeed, that is part of what it is for them to be ‘at stake”’ (Rouse, 2002, p. 25).*

In a practice-specific situation something is at issue *because* the interactants provide the context for the other one which is intelligible for the concrete other one or a relevant (in the sense of being familiar with the specific practice) community:

*“[O]ne agent’s situated environment and the possibilities it affords incorporate the activities of other agents as partially reconfiguring their shared surroundings. There is something at stake in intra-action with other agents, because its outcome shapes the intelligible possibilities for action and self-understanding by everyone involved” (Rouse, 2002, p. 21)^12^.*

Someone who does not inhabit the practice of “posting” is not able to disclose similar Gestalts on pictures in forums or blogs affectively as someone who does. Someone not being fan of a “youtuber” (or even being unfamiliar with the existence of youtubers or the possibility of them being idols) is not able to disclose the Gestalt a fan discloses via being euphoric.

The interim result is that the context of a dynamically unfolding affective situation can be described as a specific “little world.” The meaning which is disclosed in the form of complex Gestalts is co-constituted through the concerns of the involved feeling persons in relation to the practice. Life-form specific affordances affect us due to the incorporation of practice relevant norms and are thus always already meaningful and normatively structured with respect to practices and forms of living. Humans are “skilled” to disclose practice-specific normativity affectively and this skillfulness concerns the maintenance of the practices, the maintenance of specific “little worlds.”

## Situatedness II: Diachronic-Global Perspective

The synchronic situated perspective corrects an individualistic and decontextualized account of affective intentionality *spatially* by considering the concrete local environment of the feeling person. The diachronic perspective allows additionally to address an “*intertemporal*” dimension. Taking situatedness seriously now requires an integration of these perspectives in order to bring to light that, and explore how, affective intentionality in concrete encounters is structured not only by the people and artifacts present in that moment, but additionally by *the sedimented affective biography* which manifests in the practice and life form specific *emotion repertoire* a person acquires. The *emotion repertoire* is the set of meaningful Gestalts being available in a certain situation, given the learning history of the meanings of affectively relevant situations or cues (for a detailed account of emotion repertoires see von Maur, 2018, Chapter 4). Yet, taking situatedness seriously requires us going even further and considering a *global* dimension as well: Affective biographies differ depending on the era and culture in which they take place – namely the “cultural emotion repertoire.”

I firstly illustrate the diachronic dimension by taking emotional ontogenesis as one important sequence of the affective biography and by combining insights from social psychology (Parkinson et al., 2005) and philosophy (de Sousa, 1987). Already in the early stage of the affective biography, the ways of interacting with people and materiality structure life-form specifically how the world is affectively disclosed. From the beginning, the learning process of emotional meanings is a relational one: in face-to-face affective encounters, caregiver and child each react reciprocally to the gestures, facial expressions and vocalizations of the other. Through the specific feedback the child learns to ascribe meaning to the consequences of its behavior and ultimately to use it (which it initially unreflectively did) strategically. An illustrative example for this is called “coregulated behavior” (Parkinson et al., 2005, p. 237): the caregiver strongly holds the child in their arms such that it cannot move its own arms anymore. A successful coordination between the two would consist in the child trying to free its arms which causes the caregiver to lose their grip. If this does not happen, the child will experience frustration which can be interpreted as an early instance of anger. It learns to connect the whole situation of its frustrated need and the non-reacting caregiver with the resulting feelings which it will later identify and denote as anger. Such interaction contexts in which children learn to associate their reaction as an expression of particular emotions are what de Sousa (1987) calls “paradigm scenarios.” In the context of a paradigm scenario, the instinctive reaction of a child to a stimulus becomes part of an emotion. Smiling or crying for instance will become an expression of joy or anger (de Sousa, 1987, pp. 285–286). The whole complex structure of emotions (intentional object, formal object, expression, etc.) is acquired, according to de Sousa, in a paradigm scenario. *Which* strategies and behavioral patterns a child acquires and uses continuously is dependent, according to Parkinson et al. (2005), on how the caregiver interprets the behavior of the child and how they react accordingly. A screaming newborn might be perceived by one person as being legitimate in its needs, whereas another person may interpret the same affective comportment as an expression of illegitimate stubbornness. Each will react differently to the child – and thus differently shape its emotion repertoire. In the first case it is likely that anger will be used as a means to have influence in interpersonal relations. In the second case it is more likely that anger will be recognized as a potential source for conflicts and thus only be expressed if the other one will not cooperate. The way in which the caregiver handles the perceived situation of the child is itself dependent on the resources which are available in the specific socio-cultural context of the person (Parkinson et al., 2005, p. 238). Even if the frustrated needs of the child are perceived as being legitimate, the necessary resources might be missing which would allow the fulfillment of its needs. Or the child is perceived as not being justified in their needs, but the caregiver does not see any other option to calm it down than by acting according to its will. Thus,

*“[c]ulture affects the early consolidation of emotional responses at both an ideological and practical level. [.] Infants adapt to a preexisting social world, but do not simply soak up its influences like sponges. Instead, they negotiate ways of making practical or communicative use of whatever cultural resources are at hand” (Parkinson et al., 2005, p. 238).*

In a further developmental stage, emotions are not merely directed at the environment but can also have the relationship with a caregiver or object as an object. Typical phenomena of this stage of “secondary intersubjectivity” are joint attention and social referencing (ibid., p. 242). According to a study by Hornik et al. (1987), cited by Parkinson et al. (2005), 12 month old infants play less with a toy if the mother expressed disgust toward it before than in cases where the mother smiled or behaved neutrally. The infant thus seems to understand the caregiver’s evaluation of other persons or objects. The meaning of such a situation – and thus the meaning of the emotion as well as its intentional object – is structured through the concerns of the child and the caregiver against the background of the shared practice, the “little world” that both enact together; and this practice-related relational aspect enters into the constitution of meaning of the emotion-object pairing getting a place in the emotion repertoire of the child.

In a community in which relevant linguistic conventions are shared, the growing child is eventually able to use symbols in order to influence others. Objects of emotions are thus no longer restricted to the present situation but can also be abstract or anticipated aspects. Such abstract meanings are highly dependent on the socio-cultural context. The enormous influence of the permanent confrontation of media-circulated advertisement on the development of the emotion repertoire of a child is especially remarkable here. Products acquire a place in a narrative -- for instance in advertisement spots in the TV, in serials or movies, on posters, packages of sweets -- which affect children in very specific ways. Following the theory of paradigm scenarios, the affective experience is connected to the meaning that this media representation delivered^13^.

*“Children do not even need to be directly exposed to this propaganda for the cultural message to filter through to them through social networks, shaping their desires, and satisfactions. Furthermore, the stickers, badges, costumes, and play-figures that are purchased for them convey messages about group membership that also carry emotional power” (Parkinson et al., 2005, p. 244).*

In practices, these emotional evaluations materialize themselves by the social environment dealing with the products in a specific way. Take friends in kindergarten or school who wear a certain kind of clothing, possess specific games, or know the relevant music. These are as formative as the attitude of the parents with these things – prohibitions, consent, or critical utterances with respect to said objects shape the affectively disclosed meaningful Gestalt of the children. Again Parkinson et al. (2005) emphasize that children are no “cultural dupes” who blindly adopt anything their environment offers to them, but are able to use the available resources in accord with their concerns. Against the background of what I developed so far, this assertion seems to be too optimistic: the fact that possessing specific products is decisive for whether a child in kindergarten, school, or sports club belongs to the group or not is affectively effective to such a great degree that I can hardly imagine a child being able to defy. My formulated thesis above is that the “skillful dimension” of affective intentionality can be understood as the ability to disclose practice-relative “appropriate” normativity. Applied here, this would mean that a negative emotion with regards to life-form specific positively coded objects would be an explicit distancing from the norms relevant for a maintenance of this form of living. Yet, this *is* possible and even necessary in some cases, as I will illustrate in section “Situatedness III: Forms of Living Within the Subject: Normative Assessment of ‘little worlds.”’

To bring together the developed pieces so far, consider the example of Alex once again. Alex is affectable by the stone blocks the way they are because of their affective biography and the resulting emotion repertoire. Conceive for instance another person, say Elli, who, contrary to Alex, is affected by the stone blocks with pure horror and sadness. This is due to *her* emotion repertoire: during her affective biography she, as the grandchild of a Holocaust survivor, has very sensibly been brought up with the relevant material and the respective meanings -- in this case, the Holocaust memorial in Berlin, the meaning of which Alex does not know (accidentally). Alex must not have been in such a direct contact with the Holocaust herself in order to be affectable in the way Elli is. The claim here rather is that the different meaningful Gestalts being disclosed with respect to one and the same materiality cannot be understood properly by merely looking at the present moment. We need to take the diachronic dimension into account which is itself also a product of specific socio-culturally contingent circumstances. This ‘‘global’’ dimension of situatedness makes visible that also ‘‘*cultural* emotion repertoires’’ which differ between space and time need to be considered. For instance, my grandmother would not have been able to be affected by *anything* as being ‘‘Instagram-able,’’ for the form of living of ‘‘posting’’ did not exist in the first place^14^.

For the purpose of this paper, I will briefly demonstrate the operative efficacy of this dimension by considering how, for instance, different norms *about* emotions, belonging to cultural repertoires, shape the very act of affective disclosure. Importantly, cultural specificity does not (only) denote the difference between countries, nations, or continents but refers more encompassingly to shared systems of meaning that are anchored in specific socio-cultural milieus. “Culture” is understood accordingly as “learned systems of meaning, communicated by means of natural language and other symbol systems, having representational, directive, and affective functions, and capable of creating cultural entities and particular senses of reality” (D’Andrade, 1984, p. 116). Such norms for feelings direct the (affective) comportment of feeling subjects more implicitly than explicitly: internalized “cultural models” (Mesquita, 2007; Mesquita and Leu, 2007) guide the subject in identifying emotion-specific norms and demands in specific socio-cultural settings:

*“Cultural models represent not just the normative, but more importantly the habitual; they lend meaning to our daily behavior. […] The functionality of emotions within a socio-cultural context requires that they be coordinated with the specific cultural models” (Mesquita, 2007, p. 411).*

Such operationally effective cultural models especially manifest themselves in narratives *through which* one’s own emotions, and those of others, are interpreted. This results from the specific way in which the person learned to talk and think *about* emotions – as a part of the relational process of affective biographies in which emotion meanings are learned through paradigm scenarios and then are picked up, changed and transformed throughout the course of life. For instance, the ideal of humans as self-determined rational individuals which are able to control their emotions in order to supposedly clearly, “cold-bloodedly,” and factually make judgments and achieve knowledge is an example of a shared emotion culture (or even ideology) shaping the affective Gestalt disclosure of a given situation. This culture-specific narrative structures the interpretation of emotions only to the degree in which it has been acquired through the culturally situated affective biography. Think of the widespread assumption about the nature of emotions according to which there is a tension between their overwhelming power and the possibility of autonomous control. This assumption delivers a blueprint for interpreting one’s feelings (retrospectively), for how they are spoken about and – this is the most interesting thesis – how they are experienced in the very moment of taking place. A subject then for instance interprets their outrage while driving the car – to come back to the example of section “Situatedness I: Synchronic-Local Perspective” – already in the moment it is happening, and not only retrospectively *through* the narrative which developed during her affective biography; namely, that the emotion overcomes them and that they actively need to control it to supposedly be “rational” again. Thus, the labels which a person can use in order to denote the experience of an emotion are not *prior* to the emotions and are *then* added to specific episodes of experience – like a post-it, as Sara Ahmed (2010a, b) formulates this insight. Rather, the labels shape the emotional experiences themselves^15^.

It is important to note here that the affective life of humans is not determined by one static emotion repertoire referring to one specific form of living. The diachronic dimension sketched here is meant to highlight the temporal plasticity of the affective dispositions of individuals. Emotion repertoires thus have to be conceived of as malleable and constantly changing. Also, a person even might have conflicting Gestalts at hand to be disclosed in the same moment – think of the tension experienced when you do not know yet whether you want to laugh or cry about someone telling you about a mistake you made. You might disclose the “little world” of being offended or the one of being thankful for the help. The notion of meaningful Gestalts and of “little worlds” entail the plasticity and the complex nature of ways of being in the world in specific “worlds.” Humans can also “travel between worlds,” as Lugones (1987) importantly discusses. As a politically relevant aim, she conceives of this as a needed capacity in order to understand the experience of others. Understanding other “little worlds” as well as questioning one’s own, and dropping some in favor of others, are important capacities humans need to cultivate. This seems to be especially difficult, for the very mode in which these are operative is tacit and not explicitly reflected upon – as Al-Saji says with reference to Linda Martín Alcoff, “we see through our habits; we do not see them” (2014, p. 138).

We can see at this point that humans learn in socio-cultural feeling cultures to be affected and to affect others in specific ways, to ascribe meaning to these performances (by themselves and others), and to construct their current affective reality on the basis of this learning history. Thus, not only is a particular context always already normatively structured relative to socio-cultural practices, but the person themself is pre-figured in their specific affective “I can” (Al-Saji, 2014, p. 189). I will illuminate this perspective of a kind of “inverted situatedness,” a consideration of the “environment within the subject” along with all its decidedly moral, political and societal implications in the final section.

## Situatedness III: Forms of Living Within the Subject: Normative Assessment of “Little Worlds”

The subject situated in a context that is structured by a certain form of living and discloses a “little world” (with others) is a “product” of their affective biography: sedimented emotion repertoires restrict the space of possibilities for potential ways of being affected and affecting others. Yet, *individual* emotion repertoires are not only shaped by encompassing temporally and spatially specific *cultural* emotion repertoires but are even “socially extended” (Gallagher, 2013) or “invaded” (Slaby, 2016) by social structures: sociality is internalized and embodied in the subject’s (affective) comportment. Forms of living do not shape the subject from the outside but are, in a sense, already *within* the subject. Exceeding the awareness and control of the subjects, forms of living thus make up their “little worlds” – sometimes even in ways conflicting with norms and values, and ultimately ways of being-in-the-world, that the subject would reflectively endorse. The concept of “situated affective intentionality” that I established in the previous sections allows us to deepen and illuminate the concept of shared “little worlds” from a decidedly normative perspective: the concrete realities being affectively brought into existence can now be made subject to normative assessment. The critique made possible here is at the same time potentially emancipatory in its epistemic dimension by making the subject aware of the tacit structuring of their world-disclosure. My “multidimensional situatedness framework” thus provides the ground to assess the appropriateness of emotions in a much deeper way than established accounts (i.e., fittingness, moral aptness or prudence; see Deonna and Teroni, 2012 or D’Arms and Jacobson, 2000 for an overview) – namely as one that is in the end evaluating different forms of living which specific emotions support or prevent.

In the context of theories of situated *cognition*, Shaun Gallagher (2013) claims that we need to adopt a political and critical perspective on phenomena within the research of situated cognition (and affectivity, as I will argue in the following). He suggests a “liberal interpretation” of the thesis of a socially extended mind, which goes beyond the classical examples of notebooks as potential extensions of memory functions. Gallagher claims that specific social practices (for instance, manipulating the decision-making process of people who should donate at charity events) structure cognitive processes, and that the mind is in this sense *socially extended*. The crucial point is, according to Gallagher, that we can easily imagine cases in which such a socio-normative structuring of mental processes is *not in the interest of those involved*. Against the background of this assumption, he pleads for a “critical twist” in existing research in cognitive science about the thesis of the social extension of the mind (ibid., also see Gallagher and Crisafi, 2009). This results in a wide-ranging change in perspective that I suggest adopting for situated affective intentionality. Such a change does not mean merely adding more or other factors as potential extensions of the mind, but rather the interest of investigation toward the epistemic object “affectivity” changes. Not only are the operative processes or questions about the location of emotions (in the head, in the body, in the environment) the subject of investigation, but rather, socio-material factors of lifeworld practice are to be considered in their structuring role (which is potentially subject to criticism).

One domain of practice for highlighting this perspective shift is the workplace. Criticizing the functionalist paradigm of situated accounts of cognition and affectivity, Jan Slaby (2016) analyses how the minds of white-collar workers are, as he calls it, “invaded” by culturally specific technical infrastructures or institutional practices. Slaby makes clear that the unquestioned idea in the paradigm of situated cognition and affectivity (that I called functionalist, and he denotes as the “user/resource model”) runs the risk of overseeing structural effects which go beyond the personal grip as well as a one-sided positive utilization of environmental structures. To illustrate the perspective of an invasion of practice-specific affectivity into the individual, Slaby asks the reader to imagine themself to be an intern on their first day of the job in a big company. The intern finds themself in an environment in which the colleagues talk to each other and behave in a way which is unfamiliar for the newcomer. This circumstance demands to learn more than the regular ways of working. In order to belong to the company, it is not sufficient to know how to do the job but to understand which ways of comportment in which manners and circumstances are appropriate and necessary – especially of the informal kind. To “become one of them” means first and foremost, Slaby argues, to get used to *affective comportments* and *affective styles* and to adopt them (ibid.). Such a process of habituation leads to the whole way of comportment becoming second nature such that the intern does not perceive them anymore *as* practice-specific demanded affective requirements and norms. What characterizes areas of “in-depth affective modulation” in general, such as corporate work spaces, is that they at the same time demand and lead to severe shaping effects on the personality, including affectivity, which “is profoundly framed and modulated so that the affective and emotional dispositions of an individual squarely fall in line with the interaction routines prevalent in these domains.” (Slaby, 2016, p. 2) Crucial questions which are almost completely missing in the recent literature on situated affectivity^16^ can be addressed and investigated in the context of my multidimensional approach. In which ways are such formative social domains operative, how do individuals become used to them, how do comportment and affective styles mix with these life-form specific processes? All these questions have a normative implication and open up much deeper reflection on the appropriateness of emotions than most accounts deal with. Taking up the example of the “little world” which is disclosed in an office illustrates the difference between the classical situated paradigm and my approach. Interactive technologies in this area (as environmental scaffolds) lead to the enlargement of working hours in areas which have been off times before (Slaby, 2016, p. 9) – “for instance, when office workers tend to be online and available for work-related communication night and day, no matter whether on weekends or during holidays” (ibid.). Individuals thus often do not actively decide in which way the environment modulates their affectivity, and these unconscious structuring effects invading from outside often even diametrically oppose the concerns of the feeling person. The “little worlds” which are established by life form specific affective intentionality are thus not neutral and equally preferable. There are worlds we should and worlds we should not disclose – dependent on the ways we aim to be in the world more generally. Highlighting the potentially negative impact of structures and practices on affectivity, the approaches of Gallagher and Slaby suggest that something from outside invades the subject, that something concrete intends to elicit specific processes within the individual. But driving cars, being a fan of a pop group, following food trends, or giving a talk at an academic conference are practices in which the specificity of the form of living structures the character and content of affective intentionality systematically, without being intended either from inside or from outside (as in the case of charity and seeking donation, in which the structuring of cognitive processes is intentionally aimed at). These structurings are *performed* – they become real by the fact that concrete individuals affect and are affected in a specific way. The discussed practices making up the form of living of “posting,” the practices in office workplaces, as well as cultural standards about how to drive cars, already demonstrate this.

Even deeper though, our seemingly fundamentally personal desires are shaped by life-form specific practices. Thus, *which* “little worlds” we disclose is neither pure coincidence nor a solely private affair. Instead subjects *learn* for instance “what makes them happy” (Ahmed, 2010a,b) in their culturally specific and thus contingent affective biographies. According to the common picture we think that we are happy *because* that to which our happiness is directed is good. Contrary to this, Ahmed writes:

*“[R]ather than say that what is good is what is apt to cause pleasure, we could say that what is apt to cause pleasure is already judged to be good. […] Certain objects are attributed as the cause of happiness, which means they already circulate as social goods before we ‘happen’ upon them, which is why we might happen upon them in the first place” (2010a, 41).*

Thus, which ‘‘little worlds’’ appear to be attractive and thus are likely to be disclosed (together) affectively is fundamentally life-form specific: we ‘‘know’’ that champagne ‘‘tastes good,’’ that wealth ‘‘makes us happy,’’ and we associate our feelings with these objects according to this knowledge, according to the incorporated taste^17^. To drive a Porsche or SUV, to be “rich and famous,” to possess the newest iPhone, or to wear the hippest fashion label, are in the same way already marked as objects of happiness *practice and life form specifically* – just as liking oysters, listening to the opera, or reading world literature are classified as “good taste.”

For a normative assessment of emotions which takes situatedness seriously, the important implication is that not all emotions exhibit the value of “making happy,” and thus the promise of happiness guides life in *certain* directions and not others. As an “emotional community” (Rosenwein, 2002) a family, like the work place, provides specific emotion repertoires, and refuses others. “Little worlds” are brought into existence, manifested, and transformed through affective dialogical practice. According to Ahmed, the family is not an object that is associated with happiness because it actually makes us happy but because the family is classified as a good, as an object to which positive affect sticks. To be loyal to the family goes hand in hand with the expectation of happiness. This orientation toward the object “family” influences the comportment extensively: “[Y]ou have to ‘make’ and ‘keep’ the family, which directs how you spend your time, energy, and resources.” (ibid., p. 38). In a family, specific patterns of interaction and norms allow specific affections and prevent others. If we feel happy regarding these which are associated with happiness we are aligned: “we are facing the right way” (ibid., p. 37). But:

*“We become alienated – out of line with an affective community – when we do not experience pleasure from proximity to objects that are already attributed as being good. […] We become strangers, or affect aliens, in such moments. So when happy objects are passed around, it is not necessarily the feeling that passes. To share such objects (or have a share in such objects) would simply mean you would share an orientation toward those objects as being good” (Ahmed, 2010a, pp. 37–38).*

A subject who does not assimilate herself into the prescribed, learned construction of the meaning of feelings *already thereby* destroys the happiness of the others and is responsible for potential collapses of “little worlds.” If a bad mood develops at the family table, for instance, the cause for this is seen to be the person who allegedly destroys the happiness of the family – the one who “kills the joy.” By this, happiness is destroyed in several regards, not only because the situation not to be upheld in its “chastity,” but also because the family is endangered in its status as a “happy object” – because the killjoy refuses their loyalty.

This line of thought now allows us to see that aligned (“fitting”) emotions are necessary in order to sustain specific ways of interacting, thus: specific practices and forms of living. Not to feel aligned might make it impossible for some practices and forms of living to be upheld – it might end the existence of some “little worlds” and this has to be addressed normatively when it comes to the appropriateness of emotions. Emotions become important in the way that they allow or prohibit certain ways of living to be present – for good or bad. The way that meanings of emotions are learned, and how humans behave according to them, are structured through specific practices in a much more complex way as being visible if one abstracts from the multidimensional socio-structural situatedness that I have illuminated in this paper. Concrete emotions are explainable in their specificity because they allow the feeling person to partake in a specific form of living and to maintain it. Humans do not want to be “affect aliens” but rather strive for belonging, for fitting in. Against this background, the skillful dimension of affective intentionality concerns practice-specific responsivity allowing self and others to uphold the habitual “little worlds” and life-form specific realities.

## Conclusion and Outlook

The present paper offers a framework that can address processes of (shared) meaning-disclosure in interpersonal and socio-material affective practices. In the course of their life, individuals negotiate meanings of emotions in relational affective processes with their socio-cultural environment. In accord with this, in a concrete situation a subject has *particular* Gestalts available for disclosing meaning. The meanings that objects acquire in this way are relative to forms of living and are in a crucial way at once *contingent* and *persistent*. They are *contingent* relative to the life-form specific paradigm scenarios in which an individual learns the meanings of emotions. The Gestalts an individual has at their disposal would be different if the person were raised in another epoch or culture, or if they had negotiated other meanings in relational processes with the relevant people. This means that the way in which humans are – or are not – affectable by particular affordances, and the Gestalts they can or cannot affectively disclose, are co-constituted by forms of living. These forms of living, in turn, are themselves the “products” of complex historico-cultural processes of becoming, and as such constantly subject to change. At the same time, the Gestalts an individual is or is not able to disclose exhibit a certain *persistence*: the way in which a person can be affected and affect others possesses some sort of perseverance and is often very hard to change. The way in which an individual construes reality becomes incorporated as second nature. Emotion-object pairings are dependent on the convictions about the “emotional value” of the objects which obtain in a given milieu. In this way the environment “invades” the repertoire of meaningful Gestalts – namely, how meaning is affectively construed. An emotion which seems inappropriate at first glance may actually manifest a resistance against emotional ideologies which ought to be called into question in the first place. If an investigation of affective intentionality only focuses on emotion types directed on particular objects with (un)fitting formal objects, then it abstracts from and is ignorant of *the reasons for the ascription* of these formal objects to the concrete things. As long as theorists operate with a repertoire of examples such as dogs and bears and their potential dangerousness, questions of the contingency and persistence of the meaning of “dangerousness” do not occur. But against the background of my multidimensional framework of situated affective intentionality, the assumption that this works the same way for examples like “what makes us happy” is inadequate. What *actually* makes us happy and what *should* do so is neither given by certain objects nor a question of personal preferences alone. It rather becomes comprehensible and criticizable against the background of the practices making intelligible the concerns which again explain the concrete emotions. A person is not enthusiastic about a thing like a Thermomix because of its supposedly objectively valuable properties nor because of their private preference of making any meal by heating and simultaneously mixing ingredients. They do so because they practice a specific way of “how one cooks” belonging to a certain form of living guided by a socially shared narrative – in contrast to people who do not cook at all or for whom cooking is a craft. In this paper I have dealt with emotions *in the life world practice*, with emotions beyond basic forms of trigger responses, with phenomena it makes sense to consider from a situated perspective. To take situatedness seriously means to explicate how life-form specific factors systematically structure the characteristics and content of affective phenomena and the “little worlds” thus brought into existence. Hence, concrete instantiations of affective phenomena are at the same time *producers* as well as *products* of socio-culturally specific practices and forms of living – and have to be normatively assessed as such. It is not only but especially vivid when looking at forms of living being guided by transphobic, racist, sexist, or any other discriminatory emotion repertoire, that it matters *which* forms of living we sustain. The multidimensional framework developed here aims at contributing to and calling for a decidedly politically engaged situated approach to affective intentionality. It should provide the ground for a deeper analysis and a normative assessment of the effects of concrete practices and forms of living for our well-being, for what we deem to be lives worth living and for the political spaces we provide. It makes a huge difference *which* “little worlds” we disclose together affectively, and we need to direct attention to the severe and encompassing impact of this way of world-making.

## Data Availability Statement

The original contributions presented in the study are included in the article/supplementary material, further inquiries can be directed to the corresponding author.

## Author Contributions

The author confirms being the sole contributor of this work and has approved it for publication.

## Conflict of Interest

The author declares that the research was conducted in the absence of any commercial or financial relationships that could be construed as a potential conflict of interest.

## Footnotes

1 I do not claim this holds for any emotional reaction. There are also instantiations of emotions for the explanation of which this account does not help. For instance, very basic forms of trigger responses, like being afraid in front of a dangerous animal or being disgusted by rotten food, might be explained without reference to forms or living and seem to be better explained with reference to biology.

2 This is also reflected upon in the work of Matthew Ratcliffe, 2008 and a crucial facet of what he calls “existential feelings.” These are the conditions of the possibility for concrete emotional episodes to occur in the first place and thus structure the very space of possibilities for affectivity (see also Slaby, 2008). As Ratcliffe highlights that affective meaning making needs to be considered in a temporally extended manner it would be worth further studies to examine the socio-cultural structuring of existential feelings as well. For a practice-specific account of pre-reflective affective intentionality that builds upon a combination of Merleau-Ponty’s normative notion of “being *toward* the world” and Heidegger’s emphasis on the affective nature of Dasein see von Maur, 2018, chapter 2.

3 She says a “world” might be the “dominant culture’s description and construction of life, including a construction of the relationships of production, of gender, race, etc.,” (1987, p. 10) of for instance an actual society. It must not be of a whole society though but can also be “a construction of a tiny portion of a particular society. It may be inhabited by just a few people” (ibid.).

4 I use this term in connection to Ludwig Wittgenstein’s (1953) “form of life.” This concept and also his work on blind rule following importantly highlight the pre-reflective nature of norm guided behavior. Also, Martin Heidegger’s (1927) differentiation between unarticulated general understanding (*Verstehen*) and explicitly grasping (*Auslegen*) emphasizes that comportment is related to norm-guided practices but that following such norms is not a matter of reflection and deliberate action – to grasp hammering you already have to understand the general practice of carpentry (cf. Rouse, 2007, p. 643). Wittgenstein and Heidegger count as precursors of what later has been called practice theory (cf. Schatzki et al., 2001). Especially with his hermeneutics of Dasein, Heidegger influenced many authors working on humans as practically engaging, understanding beings-in-the-world. This implies a critique on individualistic, rationalistic, or representationalist ideas of human behavior (cf. especially the work of Charles Taylor and Hubert Dreyfus).

5 For a detailed account on “skillful coping” in this manner see especially the work of Hubert Dreyfus who also relates this to (background) practices (cf. Dreyfus and Wrathall, 2017). In his work as well as that of Charles Taylor (and their collaboration), also the epistemic picture influenced by Gestalt psychology (basic perception as being already meaningful) is a key issue (cf. Taylor, 2006). Both accounts as well as my approach developed here stand, in this regards, in theoretical debt to Martin Heidegger’s hermeneutics of Dasein.

6 For a detailed account on how emotions can be conceived of as following scripts see Eickers, 2019.

7 See Malafouris, 2013 or van Dijk and Rietveld, 2017 for different approaches to explore the socio-material context and its impact on intentionality.

8 The concept by Merleau-Ponty allows to see that gaining a new habit means to change one’s body schema. For instance, a blind man’s stick is integrated into the body schema: the blind man experiences the environment *via* the stick, they incorporate the stick and thus acquire the skill to inhabit the world in a different way than before (Merleau-Ponty, 1945, p. 176).

9 “The *affordances* of the environment are what it *offers* the animal, what it *provides* or furnishes, either for good or ill. […] I mean by it something that refers to both the environment and the animal in a way that no existing term does. It implies the complementarity of the animal and the environment. […] They are unique for that animal. They are not just abstract physical properties” (Gibson, 1986, p. 127).

10 This hypothesis can be opposed from the very perspective from which I build it up. Especially *such* forms of living which are in a special way hip and fashionable, one could argue maliciously, lead to the perception of very *similar* Gestalts. From a critical perspective one has to consider the socio-culturally structured affectability and meaningful Gestalts in the mode of life of “das Man,” as Heidegger calls it (see von Maur, 2018, chapter 4 for a detailed approach).

11 I develop the concept “habitual affective intentionality” in order to explain in which way emotions are relevant for the epistemic goal of understanding within socio-culturally specific contexts. The concept allows to integrate the world-directedness, the situatedness, and the habitual dimension of affective phenomena. According to my account, the specificity of a concrete instantiation of affective intentionality is an irreducible way of world-disclosure, structured through socio-cultural embeddedness and through individual habitual “orders of feeling.” Emotions, understood this way, are potentially defective for understanding processes because the habitual dimension can foreclose alternative ways of understanding and because it binds individuals to orders of feelings, allowing them to sustain their forms of life. Making these mechanisms visible allows us to think differently about potential solutions in order to overcome serious epistemic problems in everyday encounters.

12 Rouse adopts this concept from Karen Barad (1996), who introduces it in order to avoid the implication of the term “interaction” that there are two definite and confined systems or individuals (cf. Rouse, 2002, ch. 8). For the same reason, Dewey and Bentley (1949) speak about “*trans*actional” rather than “*inter*actional,” in order to avoid substantialist connotations of static entities (cf. Burkitt, 2014, p. 19). More recently, Shannon Sullivan (2001) takes up this notion in order to highlight the dynamic, co-constitutive relationship between organism and environment.

13 In this way of learning the meanings of emotions, the reciprocity highlighted by Parkinson et al. (2005) is distinctly restricted. The potential to affect that media exhibit does not only have a huge impact on children. Desires and affections are not only awakened (as if they have been present before and only need to be activated), but are rather brought into being in the first place. Often this has little to do with actual needs of the consumers. To escape this (affective) power is very hard to imagine in cases in which the individual has not developed a critical, distancing and reflexive stance toward consumism. For a detailed critique of media such as TV and their impact on (the affective repertoire of) children see for instance Bernard Stiegler’s work on “taking care” (original “prendre soin”) which highlights the need not to let alone children while consuming media and count on their alleged ability to resist. He pleas for a need to take care of them, meaning *inter alia* to teach a critical engagement with media (e.g., Stiegler, 2010).

14 The idea that there are not only individual but also cultural emotion repertoires restricting the individual ones is for instance reflected in the concept of “emotional regimes” by William Reddy (2004), Barbara Rosenwein’s (2002) concept of “emotional communities,” or Raymond Williams’^1977^ “structures of feelings.”

15 See also Reddy, who, adapting the speech act theory of John L. Austin, talks about “emotives” (2004, p. 128): “A type of speech act different from both performative and constative utterances, which both describes (like constative utterances) and changes (like per-formatives) the world, because emotional expression has an exploratory and a self-altering effect on the activated thought material of emotion.” In the same manner, Hochschild (1983) emphasized that not only the expression of emotions but also the experience of an emotion is shaped by societal convictions and norms about feeling(s).

16 For a recent exception see Haq et al., 2020, who analyze radicalization processes through the lens of situated affectivity by making use of Slaby’s concept of mind invasion.

17 In the sense of Pierre Bourdieu (1979) who uses this to refer both to gustatory and aesthetic abilities being related to different habitus.
